# Recruitment of the multiple sclerosis cohort within the European Mobilise-D clinical validation study—lessons learnt, baseline demographics and clinical characteristics

**DOI:** 10.1186/s13063-025-09404-6

**Published:** 2026-01-17

**Authors:** Gavin Brittain, Ellen Buckley, Vita Lanfranchi, Mike Long, Thanos Tsaktanis, Veit Rothhammer, Clint Hansen, Klarissa Hanja Stürner, Walter Maetzler, Lynn Rochester, Lou Sutcliffe, Isabel Neatrour, Beatrix Vereijken, Joren Buekers, Judith Garcia-Aymerich, Sarah Koch, Claudia Armengol, Heiko Gassner, Carl-Philipp Jansen, Daniel Rooks, Letizia Leocani, Giampaolo Brichetto, Gloria Dallas Costa, Clemens Becker, Giancarlo Comi, Basil Sharrack

**Affiliations:** 1 Sheffield Institute for Translational Neuroscience, University of Sheffield, Sheffield, UK; 2 NIHR Sheffield Biomedical Research Centre, Sheffield Teaching Hospitals NHS Foundation Trust, Sheffield, UK; 3 School of Medicine & Population Health, University of Sheffield, Sheffield, UK; 4 INSIGNEO Institute for in Silico Medicine, University of Sheffield, Sheffield, UK; 5 School of Computer Science, University of Sheffield, Sheffield, UK; 6 Department of Neurology, Friedrich-Alexander-University Erlangen-Nürnberg (FAU), Schwabachanlage 6, Erlangen, 91054 Germany; 7 Department of Neurology, University Hospital Schleswig-Holstein, Kiel, 24105 Germany; 8 Translational and Clinical Research Institute, Newcastle University, Newcastle Upon Tyne, UK; 9 National Institute for Health and Care Research (NIHR) Newcastle Biomedical Research Centre (BRC), Newcastle University and the Newcastle Upon Tyne Hospitals NHS Foundation Trust, Newcastle Upon Tyne, UK; 10 Department of Neuromedicine and Movement Science, Norwegian University of Science and Technology, Trondheim, Norway; 11 ISGlobal, Barcelona, Spain; 12 Universitat Pompeu Fabra (UPF), Barcelona, Spain; 13 CIBER Epidemiología y Salud Pública (CIBERESP), Madrid, Spain; 14 Department of Sport, Exercise, and Health, University of Basel, Basel, Switzerland; 15 Department of Molecular Neurology, University Hospital Erlangen, Erlangen, Germany; 16 Fraunhofer Institute for Integrated Circuits IIS, Erlangen, Germany; 17 Geriatric Center, Heidelberg University Hospital, Heidelberg, Germany; 18 Department of Geriatrics, Robert Bosch Hospital, Stuttgart, Germany; 19 Translational Medicine, Biomedical Research, Novartis, Cambridge, MA USA; 20 Vita-Salute San Raffaele University, Scientific Institute San Raffaele, Milan, Italy; 21 AISM Rehabilitation Service of Liguria, Genoa, Italy; 22 Department of Neurorehabilitative Sciences, Casa DI Cura Igea, Milan, Italy

## Abstract

**Background:**

Multiple sclerosis (MS) is a common cause of disability in working age adults. Current clinical assessments are inadequate at disability assessment or predicting clinically relevant outcomes. Loss of mobility is an important functional disability to people with MS. Mobilise-D aims to develop, validate, and implement a digital mobility solution which measures unsupervised mobility performance across several chronic conditions, including MS, using a single wearable device.

**Methods:**

Six hundred two adults with MS, an Expanded Disability Status Scale (EDSS) score of 3.0–6.5, documented disability worsening over the previous 2 years and a 30-day freedom from relapses, were recruited across four European centres.

**Results:**

Of 1416 invited, 602 participants (42%) were recruited. Primary recruitment sources were clinicians (42%) and local registries (42%). Among 616 who declined screening, the main reasons were a lack of interest (44%), the time commitment (25%) or the travel involved (13%). Participants had a mean age of 52 years; 64% were female, with a median EDSS score of 5.0. Of those, 56% had relapsing-remitting MS, 33% secondary progressive MS and 10% primary progressive MS. Falls occurred in 58% of participants in the 12 months prior to recruitment. Of those recruited, 556 (93%) participants had valid mobility data recorded.

**Conclusions:**

The longitudinal collection of clinical and unsupervised mobility assessments will provide a comprehensive dataset, allowing for the determination of digital mobility assessments’ construct validity, predictive capacity, responsiveness, and clinical meaningfulness. Novel insights into real-world mobility that describe both walking activity and gait outcomes will be gained.

**Trial registration:**

The study was registered at the ISRCTN registry on 12/10/2020, titled “Clinical validation of a mobility monitor to measure and predict health outcomes” (ISRCTN Number: 12051706).

**Supplementary Information:**

The online version contains supplementary material available at 10.1186/s13063-025-09404-6.

## Background

Multiple sclerosis (MS) is a heterogeneous central nervous system disease with widely variable clinical and pathological manifestations. An estimated 2.8 million people live with MS worldwide; the international incidence is 2.1 per 100,000 person-years, and the prevalence is increasing [1].

MS is a leading non-traumatic cause of disability in the working-age population [2]. It profoundly affects patients’ ability to carry out their activities of daily living [3]. Impairment of gait is a significant feature in MS and has been reported to be the most important patient perceived disability [4]. Increasing levels of immobility have been shown to correlate with unemployment [5, 6], loss of income [7], rising healthcare costs [8] and most domains of quality of life [9].

Current outcome measures used in MS include the Expanded Disability Status Scale score (EDSS), the timed 25-foot walk test (T25FW) and the 12-item Multiple Sclerosis Walking Scale (MSWS-12). The EDSS is the established and most frequently utilised outcome measure in MS internationally [10] but has significant limitations: it is insensitive to change, particularly for less ambulatory people; has poor inter- and intra-rater variability; is non-linear; and is unable to distinguish between differing disabilities, disease trajectories or subtypes [10–12]. The T25FW measures the construct of ‘mobility capacity’, a snapshot reflection of the best a person can do under direct observation, but not necessarily what they can do or actually do over a sustained period or in unsupervised conditions in their daily lives. Patient-reported outcome measures of walking include the MSWS-12 [13], which measures the construct of patient ‘perception’ of what they think they can do, and requires a patient to determine an average of their symptoms introspectively, thereby creating recall bias [14]. Some of these limitations are partially mitigated in trials by using standard definitions of clinically meaningful changes or confirmed disability progression, with composite endpoints having been accepted by regulatory bodies as well [15].

Whilst mobility measurements are used in interventional MS trials, they are usually a secondary outcome measure, assess mobility capacity or mobility perception (as described above), and are geared towards evaluating treatment efficacy rather than being powered to assess whether changes in gait can detect a meaningful change in disease state [16]. Within MS treatment trials, short, timed walks measured with a stopwatch account for most cases where a mobility measure was an endpoint. Such outcome measures assess walking in a standardised environment and can only provide a snapshot of a person’s capabilities. However, mobility in the real world (mobility performance), i.e. what people do continuously on a day-to-day basis, is significantly more complex than this: it encompasses different environments, symptom fluctuation, interactions with other people, multiple tasks and social contexts.

Current interventional and observational trial designs that utilise existing outcome measures do not adequately capture representative patient populations and can lack diversity [17]. As medical consultations make up 67% of the cost of MS [18], remote monitoring may prove to be a cost-saving, innovative way of providing objective monitoring with less need for one-on-one clinician time by promoting decentralised clinical care and treatment trials.

Mobilise-D is a large European consortium which aims to develop, validate and implement a digital mobility solution across several chronic conditions, including MS [19]. A technical validation study was conducted to define a comprehensive set of procedures for the metrological verification of an inertial measurement unit (IMU) device which measures real-world walking and assessed the accuracy of digital mobility outcomes (DMOs), obtained [20–23]. These DMOs included duration, number of strides, cadence, walking speed, step/stride length, and step/stride duration on a daily or weekly average.

Subsequently, a clinical validation study assessed the capacity of these technically validated DMOs to predict global (cross-cohort) and disease-specific clinical outcomes [24]. The clinical validation study was developed in conjunction with patient and public representatives [25]. Patients’ opinions regarding the acceptability of wearable devices to measure walking were explored; patients were involved in the design of the study protocol and reviewed the patient-facing documents. A Patient and Public Advisory Group (PPAG) was formed to provide advice on protocols, mitigation plans, interpretation and dissemination of results [25].

The objective of this manuscript is to report the feasibility of recruitment to a longitudinal observational study of a wearable technology in people with MS, including recommendations for future similar studies. The baseline clinical characteristics will also be reported to establish the representativeness of the study population.

## Methods

### Study design

Recruitment was planned to last 2 years beginning April 2021. Between April 2021 and September 2022, this Mobilise-D longitudinal observational cohort study recruited 602 participants with MS and followed them up every 6 months for 24 months. Participants were recruited from outpatient and inpatient services. All clinical visits were conducted at one of four participating sites: Sheffield Teaching Hospitals (USFD), Sheffield, UK; San Raffaele Hospital (USR), Milan, Italy; University Hospital Schleswig-Holstein (CAU), Kiel, Germany, and University Hospital Erlangen (UKER), Erlangen, Germany.

The full inclusion and exclusion criteria are detailed in the protocol and can be broadly summarised as follows: age ≥ 18, a diagnosis of MS as per the 2017 McDonald criteria [26], an EDSS score between 3 and 6.5 (indicating mild to moderate disability), evidence of disability worsening in the previous 2 years, absence of an MS relapse in the 30 days prior to recruitment, and absence of significant medical illness in the last 3 months [24].

Patients’ medical notes and information provided during routine clinical consultations were screened against the eligibility criteria. If eligible and interested in participating, an invitation letter and participant information sheet were provided. Participants were given at least 1 day to read the information sheet and consider whether to participate in the study. Travel to clinical sites was reimbursed.

Demographic and clinical variables collected at baseline included birth year, gender (collected as ‘male’, ‘female’ or ‘prefer not to say’), height, weight, shoe size, leg length, education, employment, marital status, living arrangements, overall health status, smoking history, alcohol consumption, ethnicity, admission to hospital or care home within the last 12 months, and use of mobility aids. MS specific information collected included symptom onset, diagnosis date, clinician-determined disease subtype, relapse history, medication history, and non-pharmacological interventions. Clinicians assessed and patient-reported outcome measures were collected at each study visit, and source data was captured using electronic report forms by participants or study staff. Clinical outcome measures included the EDSS [27], the Multiple Sclerosis Functional Composite (MSFC) [28], 6-Minute Walk Test (6MWT) [29], Timed Up-and-Go (TUG) test [30], Short Physical Performance Battery (SPPB) [31], Low Contrast Letter Acuity (LCLA) [32], hand grip strength measured by a hand-held dynamometer and the Symbol Digit Modalities Test (SDMT) [33]. Patient-reported outcome measures included the Patient Determined Disease Steps (PDDS) scale [34], MSWS-12 [13], Modified Fatigue Impact Scale (MFIS) [35], fall events and fall-related injuries, monitored retrospectively at baseline and via monthly falls diaries thereafter, short Falls Efficacy Scale International (Short FES-I) [36], social isolation and loneliness (UCLA Loneliness scale) [37], Patient Health Questionnaire (PHQ-2), Short Mini-Mental State Examination (SMMSE) [38], Visual Analogue Scale (VAS) for pain [39], Euro-Qol (EQ-5D) [40], Groll Functional Comorbidity Index (FCI Groll) [41] and the Late-Life Functional Disability Index (LLFDI) [42].

Following the clinical assessment, patients underwent a digital mobility assessment (DMA) consisting of 7 days of unsupervised mobility monitoring using a single wearable device located at the lower back.

All statistical analyses were conducted using R Studio, version 2023.06.1. Mean, standard deviation (SD) and 95% confidence intervals are displayed for normally distributed variables without extreme outliers and median and interquartile range (IQR) otherwise. Statistical significance was set at *p* < 0.05. Between MS subtype group differences were assessed with a one-way analysis of variance (ANOVA) or the Kruskal-Wallis test, dependent on whether the assumptions of normality and homogeneity of variances were met, with post hoc analysis conducted with Tukey’s honestly significant difference (HSD) test or Bonferroni correction, respectively.

## Results

### Recruitment

**Fig. 1:**
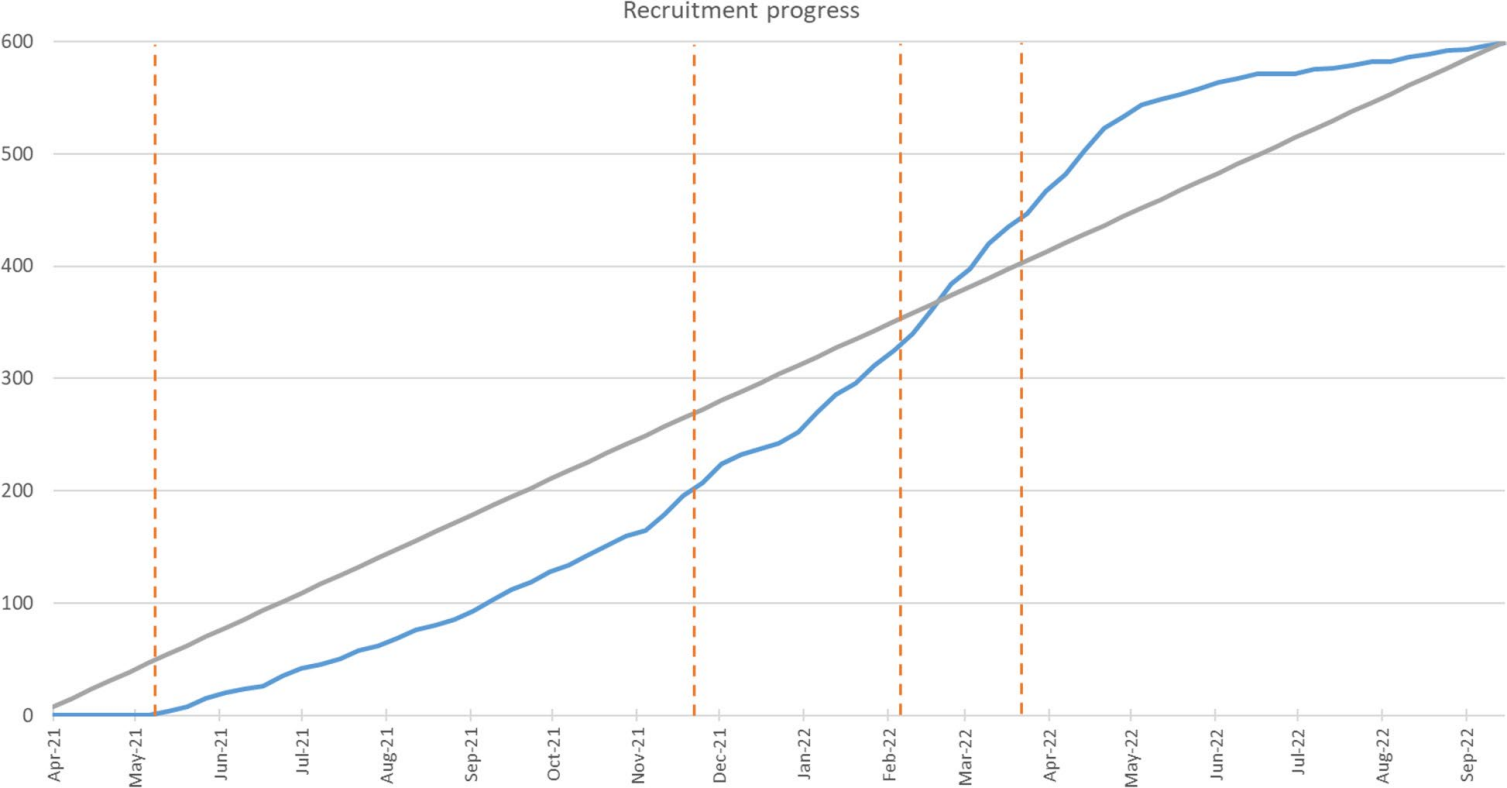
Study recruitment. The blue line indicates actual recruitment, the grey line indicates planned recruitment, and the orange dotted lines indicate each site opening

The first participant was recruited at the first site in May 2021 (Fig. 1). The Corona pandemic led to a significant delay in the opening of the second site. Mitigation arrangements led to the inclusion of two additional sites in Germany and the extension of recruitment by 1 year. Appointment reminders were additionally introduced. By September 2022, recruitment was complete: 17 (2.8%) participants were recruited in Erlangen, 45 (7.5%) participants in Kiel, 240 (39.9%) participants in Milan and 300 (49.8%) in Sheffield.

**Fig. 2:**
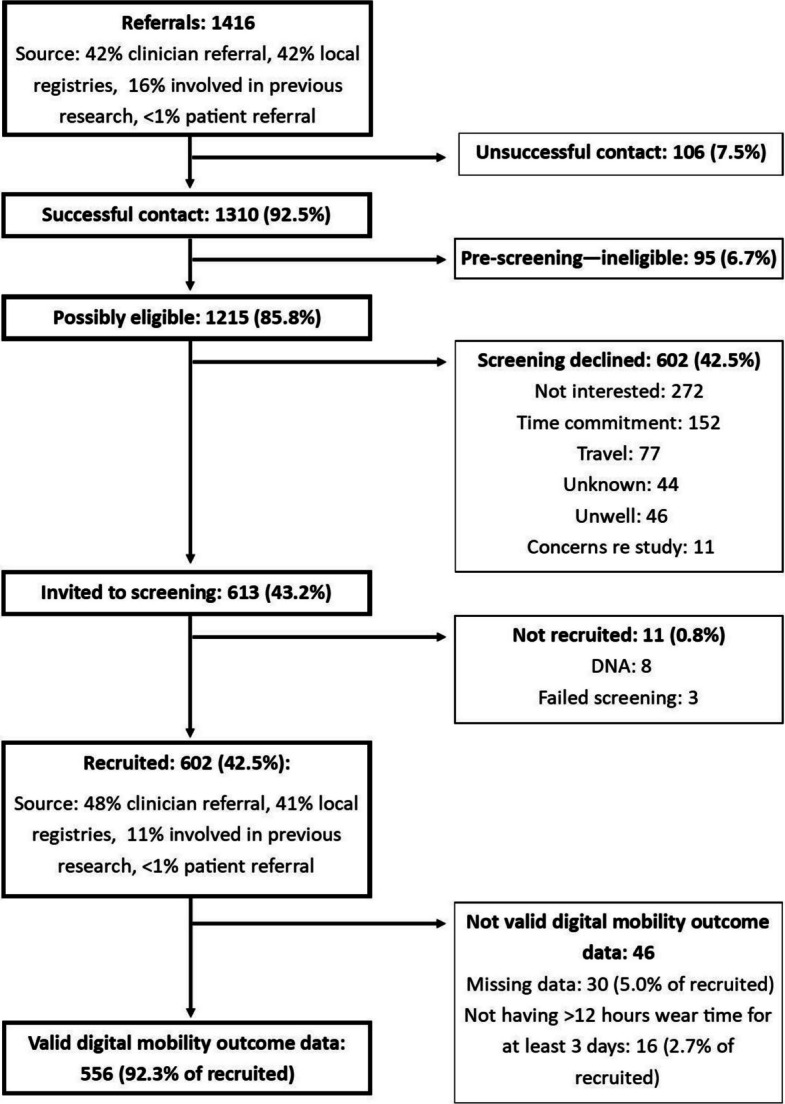
Study recruitment flowchart. Percentages calculated from the number of referrals, unless stated otherwise

Referrals (*n* = 1416) were identified from several sources (Fig. 2):

- Clinician referrals (42%) included doctors, nurses and multidisciplinary team members who had conducted a pre-screen against eligibility criteria checklists and discussed the invitation with patients in person or by telephone.
- ‘Local registries’ (42%) included patients who had previously agreed to be invited for research opportunities and electronic case note searches.
- Patients ‘involved in previous research’ (16%) included patients known to the research teams from previous observational and treatment trials who had indicated a wish for further trial involvement.
- A single patient was referred from a patient within the study.

All referrals were contacted (successful contact in 1310 cases, 93% of referrals) by telephone and pre-screened against a checklist of the eligibility criteria. Patients who appeared to fulfil the eligibility criteria (*n* = 1215, 86% of referrals) and expressed an interest in joining the study were invited to do so at a mutually convenient time.

Approximately half of those deemed potentially eligible at pre-screening agreed to undergo screening (613, 43% of referrals) and 602 (99%) of those screened were recruited. In most cases where screening was declined, this was due to a lack of interest in the study or research (45% of screening declines) or an inability to commit the required time (25% of screening declines) for the study procedures. In the remaining cases, other reasons included:

- ‘Travel’ (13% of screening declines), encompassing patients who either could not travel to the centre regardless of distance or those who felt the distance to travel was too great.
- ‘Unwell’ (8% of screening declines) included participants who were deemed eligible at pre-screening but self-reported that their MS, or an alternative condition, precluded involvement.
- ‘Concerns regarding the study’ (2% of screening declines) included potential lack of privacy of a wearable monitor, fear of family or colleagues discovering their diagnosis, and the validity of the study and sensors used.
- Eleven patients (< 1%) were invited to screening but not recruited (8 did not attend, and 3 failed screening).

### Demographics and clinical characterisitcs

Just over half, 53% (Table 1), of participants had relapsing-remitting MS (RRMS), with the remainder (36%) having secondary progressive MS (SPMS) or (10%) primary progressive MS (PPMS), according to either their clinical records or the disease-modifying treatment they received. The mean age was 52.3 years. USR’s primary source of referrals was a rehabilitation facility, resulting in the highest proportion of participants with progressive forms of MS (PPMS and SPMS) compared to other sites.

**Table 1:** Demographics of the recruited cohort, spilt by MS subtype and recruiting site

	**All**	**By subtype**			**By site**				
***n* = **	602	RRMS SPMS PPMS	320 (53%) 219 (36%) 63 (10%)			Total	RRMS	SPMS	PPMS
					CAU UKER USFD USR	45	24	10	11
						17	3	11	3
						300	205	70	25
						240	88	128	24
**Age, mean (SD, range)**	52.3 (10.8, 21–77)	RRMS SPMS PPMS	48.9 (10.8) 56.4 (8.9) 55.3 (11.1)		CAU UKER USFD USR	50.9 (10.2) 49.9 (9.4) 51.6 (11.7) 53.6 (9.7)			
**Gender (%):**			Female	Male		Female		Male	
Female Male	387 (64%) 215 (36%)	RRMS SPMS PPMS	229 (72%)	91 (28%)	CAU UKER USFD USR	31 (69%)		14 (31.1%)	
			139 (63%)	80 (37%)		9 (53%)		8 (47.1%)	
			19 (30%)	44 (70%)		211 (70%)		92 (30.3%)	
						137 (57%)		103 (42.9%)	
**Ethnicity (%)**		-	-		-	-			
Asian Black/African/Caribbean Mixed Other Not collected Not disclosed White	5 (< 1%) 9 (2%) 17 (3%) 1 (< 1%) 24 (4%) 5 (< 1%) 541 (90%)								
**Height, mean in cm (SD)**	170.2 (9.3)	RRMS SPMS PPMS	170.0 (8.8) 169.1 (9.3) 175.0 (10.1)		CAU UKER USFD USR	172.2 (10.8) 173.8 (10.1) 170.6 (8.9) 169.1 (9.2)			
**Weight, mean in kg (SD)**	76.5 (18.4)	RRMS SPMS PPMS	78.1 (18.9) 73.1 (17.7) 80.2 (16.6)		CAU UKER USFD USR	80.4 (22.7) 76.8 (13.9) 81.3 (18.0) 69.7 (16.2)			
**Body mass index (SD)**	26.3 (5.8)	RRMS SPMS PPMS	27.0 (6.1) 25.5 (5.4) 26.1 (5.0)		CAU UKER USFD USR	27.1 (6.7) 25.0 (3.7) 28.0 (6.0) 24.3 (4.7)			
**Employment:**			Working	Not working		Working		Not working	
Carer Full time Home-maker Part time Retired Sick leave Student Unemployed	2 (< 1%) 193 (32%) 28 (5%) 101 (17%) 193 (32%) 25 (4%) 1 (< 1%) 59 (10%)	RRMS SPMS PPMS	183 (57%)	137 (43%)	CAU UKER USFD USR	17 (38%)		28 (62%)	
			78 (36%)	141 (64%)		10 (59%)		7 (41%)	
			30 (48%)	33 (52%)		138 (46%)		162 (54%)	
						129 (54%)		111 (46%)	
**Educational years, full time (SD)**	14.6 (3.9)	RRMS SPMS PPMS	14.7 (3.5) 14.5 (4.1) 14.4 (4.8)		CAU UKER USFD USR	16.8 (4.3) 14.9 (2.9) 14.3 (3.4) 14.5 (4.3)			
**Ever smokers**			True	False		True		False	
True False	305 (51%) 297 (49%)	RRMS SPMS PPMS	159 (50%)	161 (50%)	CAU UKER USFD USR	32 (71%)		13 (29%)	
			105 (48%)	114 (52%)		8 (47%)		9 (52%)	
			41 (65%)	22 (35%)		144 (48%)		156 (52%)	
						121 (50%)		119 (50%)	

*Abbreviation*: *PPMS* primary progressive multiple sclerosis, *RRMS* relapsing-remitting multiple sclerosis, *SD* standard deviation, *SPMS* secondary progressive multiple sclerosis

Almost two thirds (64%) of participants were females: 30% of the PPMS group, 72% of the RRMS group and 63% of the SPMS group. The vast majority (90%) self-reported as being of white ethnicity. Just under half, 49% (full-time = 32%, part-time = 17%), of participants reported being in employment. Mean patient-reported symptom duration (Table 2), was 224.1 months (SD135.6). Annual relapse rate, in the preceding 2 years, was 0.12. Higher aid use was noted outdoors versus indoors, with 75% being unaided indoors vs 23% outdoors.

**Table 2:** Select MS disease specific descriptors, split by subtype. Between group differences were assessed with a one-way analysis of variance (ANOVA) or the Kruskal–Wallis test, dependent on whether the assumptions of normality and homogeneity of variances were met with post hoc analysis conducted with the HSD test or Bonferroni correction, respectively

All			Subtype						
									p-value
**Symptom duration in months, mean (SD)**		228 (153), 95% CI 216–240	RRMS SPMS PPMS	196.2 (129.1), 95% CI 186–222 289.7 (128.0), 95% CI 273–307 137.5 (90.1), 95% CI 115–160					PPMS vs SPMS < 0.001 PPMS vs RRMS < 0.01 RRMS vs SPMS < 0.001
**Relapses in previous 12 months, mean (SD)**		0.12 (0.4), 95% CI 0.09–0.15	RRMS SPMS PPMS	0.2 (0.5), 95% CI 0.11–0.21 0.1 (0.3), 95% CI 0.03–0.10 -					NS
**Current disease modifying treatment use or prior use of an induction approach**		276 (46%)	RRMS SPMS PPMS	160 (50%) 89 (41%) 25 (40%)					-
**Aid indoor:**	AFO/FES alone Unilateral (U) Bilateral (B) Wheelchair (W) None (N)	4 (< 1%) 79 (13%) 69 (11%) 0 450 (75%)		AFO/FES	U	B	W	N	-
			RRMS SPMS PPMS	1	30	9	0	287	
				2	43	50	0	121	
				1	6	9	0	43	
**Aid outdoor:**	AFO/FES alone Unilateral (U) Bilateral (B) Wheelchair (W) None (N)	15 (3%) 188 (31%) 137 (23%) 246 (41%) 16 (23%)		AFO/FES	U	B	W	N	-
			RRMS SPMS PPMS	7	92	33	7	188	
				4	76	86	6	44	
				3	19	16	3	18	
**Retrospectively reported falls in the last year (%):**				True		False			
True False		349 (58%) 253 (42%)	RRMS SPMS PPMS	158 (49%) 148 (67%) 43 (68%)		162 (151%) 71 (33%) 20 (32%)			PPMS vs SPMS NS PPMS vs RRMS < 0.05 RRMS vs SPMS < 0.05
**Of self-reported fallers, mean falls in the last year (SD)**		10.8 (37.4), 95% CI 6.9–14.7	RRMS SPMS PPMS	8.9 (27.3), 95% CI 6.6–10.9 9.8 (34.8), 95% CI 6.1–13.7 23.2 (68.4), 95% CI 7.6–35.3					NS
**Of self-reported fallers, reported injurious falls in the last year (%)**				True		False			
	True False	187 (54%) 162 (46%)	RRMS SPMS PPMS	94 (58%) 70 (50%) 22 (51%)		68 (42%) 71 (50%) 21 (49%)			NS

*Abbreviation*: *AFO/FES* ankle foot orthosis/functional electrical stimulator, *CI* confidence interval, *NS* non-significant, *PPMS* primary progressive multiple sclerosis, *RRMS* relapsing-remitting multiple sclerosis, *SD* standard deviation, *SPMS* secondary progressive multiple sclerosis

### Clinical observations

**Fig. 3:**
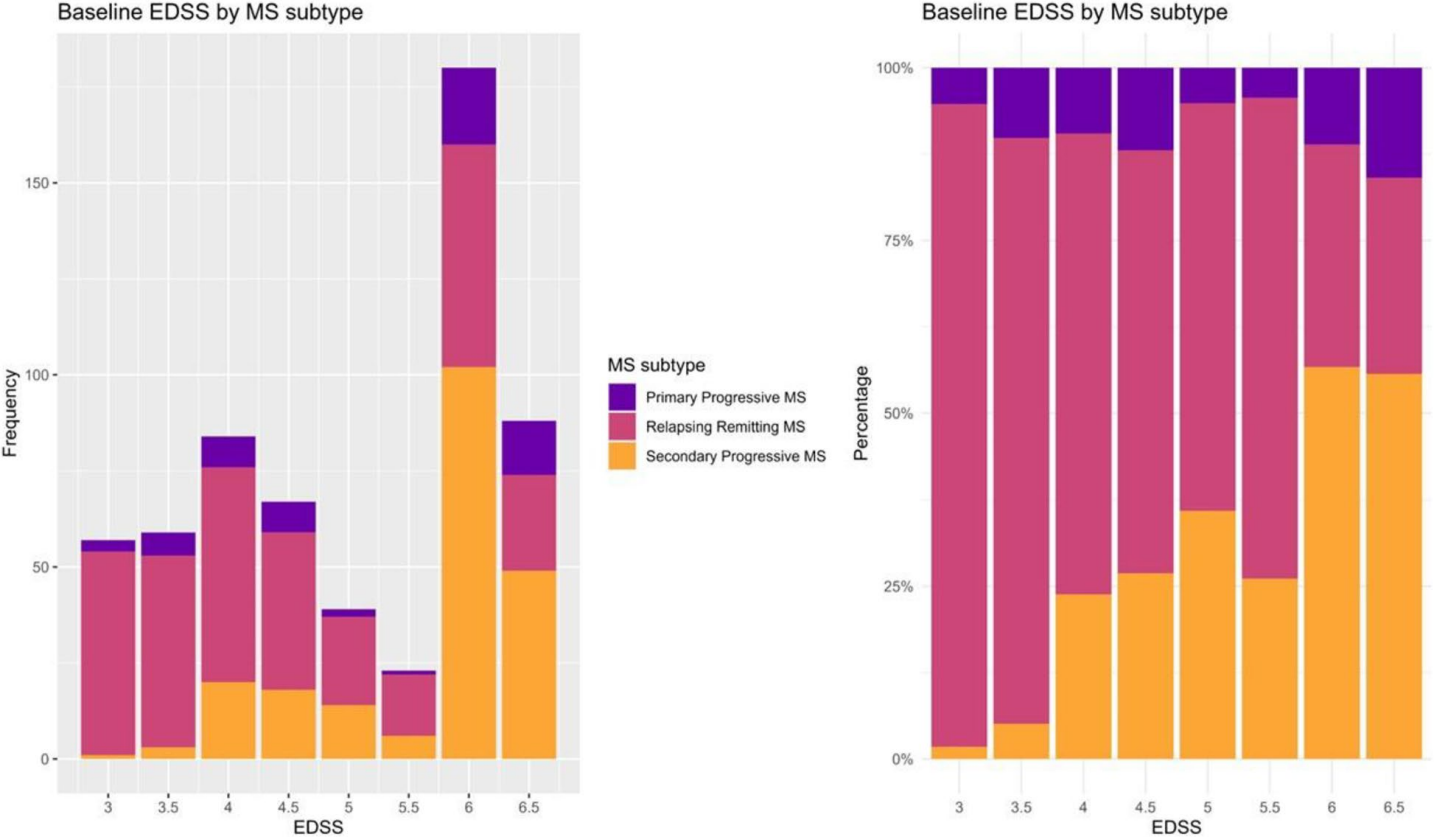
Frequency and percentage distribution of EDSS at baseline

Patient-reported falls were noted in the majority (58%) of participants in the 12 months prior to baseline. Of those who did fall, the mean was almost once per month (10.9, SD 37.5), and 54% reported injurious falls. The short FES-I mean score (Table 3) of 14 (SD 5) indicates a moderate–high degree of fear of falling. The cohort’s median EDSS of 5.0 (interquartile range, IQR, 2) indicates a moderately disabled cohort, although two peaks of EDSS were noted (Fig. 3). A third (*n *= 200) of participants had an EDSS score ≤ 4, i.e. no clinically apparent walking limitation. The mean SDMT (45.6, SD 13.7) for all participants indicates difficulties with processing speed; however, cognitive impairment was not identified via the SMMSE (mean 5.7, SD 0.6) or PASAT scores (mean 40.6, SD 14.1).

**Table 3:** Select clinical and patient reported outcome measures, split by subtype. Between group differences were assessed with a one-way analysis of variance (ANOVA) or the Kruskal–Wallis test, dependent on whether the assumptions of normality and homogeneity of variances were met with post hoc analysis conducted with the HSD test or Bonferroni correction, respectively. Abbreviation: *CI* confidence interval, *IQR* interquartile range, *NS* non-significant, *PPMS* primary progressive multiple sclerosis, *RRMS* relapsing-remitting multiple sclerosis, *SD* standard deviation, *SPMS* secondary progressive multiple sclerosis

	**All**	**Missing data**	**By subtype**		
					*p*-value
**EDSS, median (IQR)**	5 (2)	5 (< 1%)	RRMS SPMS PPMS	4.5 (2.5) 6 (1.0) 6 (2)	PPMS vs SPMS < 0.05 PPMS vs RRMS < 0.001 RRMS vs SPMS < 0.001
**PDDS, median (IQR)**	4 (3)	3 (< 1%)	RRMS SPMS PPMS	3 (3) 5 (3) 4 (2)	PPMS vs SPMS < 0.05 PPMS vs RRMS NS RRMS vs SPMS < 0.05
**9-hole peg test, mean in s (SD)**	29.4 (10.5), 95% CI 28.5–30.3	27 (5%)	RRMS SPMS PPMS	26.8 (7.6), 95% CI 25.8–27.7 33.0 (13.0), 95% CI 30.9–35.0 30.9 (10.5), 95% CI 28.0–33.8	PPMS vs SPMS NS PPMS vs RRMS < 0.001 RRMS vs SPMS < 0.001
**6MWD, mean in m (SD)**	294.3 (135.0), 95% CI 283.2 −305.4	37 (6%)	RRMS SPMS PPMS	348.1 (141.5), 95% CI 332.6–363.6 216.8 (118.8), 95% CI 201.0–232.5 277.1 (150.4), 95% CI 239.9–314.2	PPMS vs SPMS < 0.01 PPMS vs RRMS < 0.001 RRMS vs SPMS < 0.001
**T25FW, mean in s (SD)**	10.4 (8.4), 95% CI 9.8–11.1	18 (3%)	RRMS SPMS PPMS	8.0 (4.6), 95% CI 7.5–8.5 13.8 (11.3), 95% CI 12.3–15.3 11.7 (9.0), 95% CI 9.5–13.9	PPMS vs SPMS < 0.05 PPMS vs RRMS < 0.001 RRMS vs SPMS < 0.001
**SPPB, mean (SD)**	7.5 (3.0), 95% CI 7.3–7.8	1 (< 1%)	RRMS SPMS PPMS	8.7 (2.5), 95% CI 8.4–9.0 5.9 (2.8), 95% CI 5.5–6.3 7.2 (3.0), 95% CI 6.4–7.9	PPMS vs SPMS < 0.01 PPMS vs RRMS < 0.001 RRMS vs SPMS < 0.0001
**SDMT, mean (SD)**	45.6 (13.7), 95% CI 44.5–46.7	4 (< 1%)	RRMS SPMS PPMS	48.5 (13.8), 95% CI 47.0–50.1 41.7 (41.0), 95% CI 39.8–43.6 44.2 (13.1), 95% CI 41.47.4	PPMS vs SPMS NS PPMS vs RRMS < 0.05 RRMS vs SPMS < 0.001
**PASAT, mean (SD)**	40.7 (14.1), 95% CI 39.5–41.8	46 (8%)	RRMS SPMS PPMS	42.0 (16.4), 95% CI 40.2–43.8 38.6 (18.8), 95% CI 36.1–41.1 40.7 (15.9), 95% CI 36.8–44.6	NS
**SMMSE, mean (SD)**	5.7 (0.6), 95% CI 5.6–5.7	0 (0%)	RRMS SPMS PPMS	5.7 (0.6), 95% CI 5.6–5.8 5.6 (0.7), 95% CI 5.6–5.7 5.7 (0.6), 95% CI 5.6–5.9	NS
**MSWS-12, mean (SD)**	38.8 (14.4), 95% CI 37.6–39.9	4 (< 1%)	RRMS SPMS PPMS	34.0 (14.4), 95% CI 32.4–35.5 44.7 (13.0), 95% CI 43.0–46.4 42.7 (13.9), 95% CI 39.3–46.1	PPMS vs SPMS NS PPMS vs RRMS < 0.001 RRMS vs SPMS < 0.001
**Short FES-I, mean (SD)**	13.8 (5.1), 95% CI 13.4–14.2	4 (< 1%)	RRMS SPMS PPMS	12.9 (5.2), 95% CI 12.3–13.4 14.8 (4.8), 95% CI 14.1–15.4 15.5 (5.0), 95% CI 14.2–16.6	PPMS vs SPMS NS PPMS vs RRMS < 0.001 RRMS vs SPMS < 0.001
**MFIS, mean (SD)**	40.3 (18.7), 95% CI 38.8–41.8	4 (< 1%)	RRMS SPMS PPMS	41.6 (19.8), 95% CI 39.4–43.8 38.2 (17.0), 95% CI 35.9–40.5 40.5 (18.7), 95% CI 35.9–45.1	NS

Of those recruited, 556 (93%) participants had valid mobility data collected (Fig. 2). For the 46 participants not having valid mobility data, this was due to having missing data for 30 (5% of the cohort), i.e. incomplete return of the device or a failed/corrupt upload, and in the remaining 16 (3% of the cohort) the weartime threshold of ≥ 12 h for ≥ 3 days was not met [43].

A higher mean age, increased likelihood of being a man, and lower scores in most clinical and patient-reported outcome measures were seen in those with both PPMS and SPMS vs RRMS (*p* < 0.05). Smoking was more likely in those with PPMS.

## Discussion

This study represents the largest cohort of people with MS undergoing longitudinal real-world mobility monitoring using a wearable device. Recruitment was slower than expected, with a 6-month extension needed, and a high number of referrals were required to achieve the recruitment target. The conversion of potentially eligible to recruited participants was lower in this observational study than in randomised controlled trials of similar MS populations: 50% versus 55% in MS-SMART and 84% in MS-STAT2 [44]. This discrepancy is not unexpected, given the perceived lack of personal benefit from participation in an observational study. Future observational studies could attempt to increase engagement by highlighting indirect benefits, such as providing informative feedback on the study assessments. Williams et al. [44] utilised online registration portals, from which most of their referrals stemmed. However, the conversion of potential participants registered via online portals to successful recruitment was lower than that for clinician referrals, indicating that many self-referrals in such portals are required. The small number and wide geographical distribution of sites in Mobilise-D made using national and international online portals less attractive, although this option could be considered for future multicentre observational trials. In this study, local registries, which had to be specifically compiled, had an equal yield of referrals to clinician referrals, which is surprising given the clinician referrals were essentially pre-screened twice, thereby theoretically reducing the risk of screen failure. However, once compiled, they could be utilised quickly. All centres had a dedicated recruitment team with clinician input, which is known to be a driver of successful recruitment [45]. One site utilised a manual alert system for potentially eligible participants with upcoming clinical appointments. Similar automated referrals are a known driver of recruitment success [45].

Appointment reminders are known to drive recruitment success [46]. Reminder phone calls to participants on the working day before their appointment and the pre-screening of participants before the recruitment invitation, with broad eligibility criteria, resulted in 99% of participants attending screening being recruited. When a patient could no longer attend, this was rescheduled. Although details of potential participants who declined referral from clinicians were not recorded, study assessors noted that many of these participants cited concerns about the ongoing effects of the coronavirus pandemic. This concern may have introduced a bias into the cohort, i.e. those patients who deemed themselves most at risk did not participate. Considerable efforts were made to ensure that study visits were conducted in line with infection control guidelines, and relaying these principles to participants may have reduced anxiety about attending an observational trial during a pandemic.

Expected differences were seen across the disease subtypes in age, clinical history and outcome measures, supporting generalisability to an MS population. The higher mean age for participants with progressive MS is unsurprising, given (a) the time it takes for conversion from RRMS to SPMS to be clinically identified, (b) that increasing age is a risk factor for SPMS conversion and (c) the known higher age at diagnosis for PPMS [47, 48].

Across disease subtypes, a higher proportion of participants with a female sex (2.3:1) are expected to have RRMS at diagnosis, whilst male sex is a known risk factor for progression to SPMS [49], complicating the estimation of the expected gender distribution of participants. It is also likely that some of the participants were erroneously identified as RRMS, given that the inclusion criteria mandated progression over the preceding 2 years and due to a lack of clinical clarity of their disease course. Country-specific prescribing criteria also suggest stopping or switching disease-modifying treatment following a transition to SPMS, thereby dissuading a formal reclassification of subtype. Additionally, whilst there is limited observational study data, it is known that women are underrepresented in randomised controlled trials [50]. It is not unexpected, therefore, that the gender split between subtypes varies slightly from the reported prevalence of MS, with a higher ratio of women being different across RRMS (female-to-male ratio of 2.5:1) and SPMS (1.7:1) subtypes. In PPMS, an equal split would be expected, although more men were recruited (0.5:1).

The differing symptom duration found across the subtypes is as expected, given the length of time required for progression to be clinically detected. PPMS participants had the lowest symptom duration, owing, presumably, to the fact they automatically met the inclusion criteria of ‘progression’, meaning any patient with PPMS and an EDSS of 3, no matter how early in their disease course, was eligible.

### Limitations

Many barriers to participation in clinical trials and observational studies exist, and these include living in rural areas, having limited access to an MS clinic, and no reimbursement for the time or expenses during a trial [51]. The recruitment strategy, primarily driven by clinician referrals, registries created from clinical encounters, and the need for in-person rather than remote assessment, at least at baseline, may have affected trial population diversity, creating a sample biased towards those that are typically able to attend such encounters. The study inclusion criteria of EDSS 3.0–6.5 (i.e. the upper limit being the ability to walk ≥ 5 m with bilateral assistance) also excluded non-ambulant participants. The future use of digital health technologies, such as mobility monitors, when used to facilitate decentralised trials, may minimise study burden to patients and improve access [17]. These factors are a possible driver behind 38% of the non-recruited potential participants. However, a single digital health technology which concentrates on one aspect of disability, as is the case for DMOs, will not capture the heterogeneity of symptoms and the full extent of disability in all people with MS. Therefore, DMOs will likely need to be used as supplementary tools to augment alternative and existing measures.

At baseline, the cohort was, on average, moderately disabled according to their EDSS score (median = 5.0) and the differences in clinical outcome measures of various domains, such as the T25FW and the SDMT, across disease subtypes were as anticipated. The trial design, including a relatively high rate of Progressive MS participants, likely influenced the recruited population in this regard, although it was as expected. Some of the future study findings may not therefore be generalisable beyond the recruited population as influenced by the inclusion criteria, i.e. patients with lower or higher disability levels.

### Lessons learnt from recruitment in the Mobilise-D clinical validation study

- Deployment of dedicated recruitment teams with clinician engagement was essential.
- Pre-screening processes (informal and checklist-based) led to a 99% recruitment success at screening, thereby reducing resources lost to screen failures.
- Local registries, once compiled, were highly effective but required upfront effort.
- Appointment letters, or reminder calls the day before visits, improved attendance.
- Acknowledging and addressing participant concerns or anxieties around participation is crucial.
- Conversion rates were lower in this observational study than in interventional studies; potential strategies to improve this include improving engagement by highlighting any indirect benefits, such as providing participant feedback from participation.

## Conclusion

The lessons learnt in this study, primarily around the difficulty and delays faced when recruiting patients, should be taken forward into future observational and digital health trials. To our knowledge, this is the largest cohort (*n* = 602) of patients with MS undergoing detailed longitudinal clinical review and in-depth real-world mobility monitoring using a wearable device. This study additionally is the largest to collect retrospective and prospective information on falls incidence in a multiple sclerosis cohort. The Mobilise-D MS cohort appears to be generalisable to the ambulant MS population, and we anticipate that the baseline mobility and longitudinal results will lead to the development of clinically useful tools for assessing mobility and predicting clinically relevant future outcomes.

## Supplementary Information

Supplementary Material 1. STROBE checklist.

## Data Availability

The datasets generated during and/or analysed during the current study are not publicly available yet. The Mobilise-D consortium has collected a high volume of quantitative and qualitative data related to its primary objectives. In particular, comprehensive data have been collected within the cross-sectional technical validation study (TVS, 2020–2022) and the longitudinal clinical validation study (CVS, 2021–2024). The partners involved in the Mobilise-D project are dedicated to sharing the data, algorithms, and code generated during the project with the wider scientific community. In doing so, we are committed to considering and respecting the data and privacy rights of the study participants, adhering to the relevant laws, and ensuring that the needs of the Mobilise-D researchers who are writing and publishing research papers are met. Additionally, we will only release data that has been subject to a rigorous quality assurance process.
